# Temperature Sensitivity of Soil Respiration to Nitrogen Fertilization: Varying Effects between Growing and Non-Growing Seasons

**DOI:** 10.1371/journal.pone.0168599

**Published:** 2016-12-16

**Authors:** Qingfang Liu, Rui Wang, Rujian Li, Yaxian Hu, Shengli Guo

**Affiliations:** 1 College of Resources and Environment, Northwest A&F University, Yangling, China; 2 State key laboratory of soil erosion and dryland farming on the Loess Plateau, Institute of Soil and Water Conservation, Northwest A&F University, Yangling, Shaanxi, China; 3 Institute of Soil and Water Conservation, Chinese Academy of Sciences and Ministry of Water Resource, Yangling, China; Tennessee State University, UNITED STATES

## Abstract

Nitrogen (N) fertilization has a considerable effect on food production and carbon cycling in agro-ecosystems. However, the impacts of N fertilization rates on the temperature sensitivity of soil respiration (*Q*_10_) were controversial. Five N rates (N0, N45, N90, N135, and N180) were applied to a continuous winter wheat (*Triticum aestivum* L.) crop on the semi-arid Loess Plateau, and the *in situ* soil respiration was monitored during five consecutive years from 2008 to 2013. During the growing season, the mean soil respiration rates increased with increasing N fertilization rates, peaking at 1.53 μmol m^−2^s^−1^ in the N135 treatment. A similar dynamic pattern was observed during the non-growing season, yet on average with 7.3% greater soil respiration rates than the growing season. In general for all the N fertilization treatments, the mean *Q*_10_ value during the non-growing season was significantly greater than that during the growing season. As N fertilization rates increased, the *Q*_10_ values did not change significantly in the growing season but significantly decreased in the non-growing season. Overall, N fertilization markedly influenced soil respirations and *Q*_10_ values, in particular posing distinct effects on the *Q*_10_ values between the growing and non-growing seasons.

## Introduction

Temperature sensitivity (i.e., the *Q*_10_ value, which represents variations in the soil respiration rate over a temperature shift of 10°C) is an essential index to quantify and evaluate the carbon (C) cycle and the future global C balance [1–3]. It is also of particular relevance to predict the potential carbon dioxide (CO_2_) efflux feedback between terrestrial ecosystems and future global warming scenarios [4].

In specific for agro-ecosystems, variations in *Q*_10_ values are influenced by agronomic management practices as well as natural environment. N fertilization is a common field practice to improve soil fertility and crop growth, and sustain food production [5–7]. In China, arable soils are intensively cultivated through high N fertilizer inputs (23 million Mg in 2011), which account for approximately 30% of the total N fertilizer used around the world [8]. In particular, for the loess region of China, where inherent soil fertility is poor and natural N levels are particularly low [9]. N fertilization has been widely applied and considerably affect soil properties in consequences.

To be specific, N fertilization can potentially change the quantity and quality of substrate inputs [3, 10, 11], alter soil micro-environment, and further influence the composition and biological activity of soil microbial communities [12–15]. All these changes induced by N fertilization can in turn potentially affect the performance of soil respiration and its sensitivity to temperature changes. However, conflicting results have been reported in previous literature. For instance, a meta-analysis of 138 cropland experiments showed that N fertilization significantly increase soil respiration by 12.4% [16]. Nevertheless, inhibitory effect [17, 18], and no significant effect [19] of N fertilization on soil respiration were reported in other reports. Such inconsistent reports on the effects of N fertilization to soil respiration were probably caused by the complex dynamics between different components of soil respiration during different seasons.

In the case of winter wheat on the Chinese Loess Plateau, the drivers to soil respiration are significant different between growing and non-growing season. Soil respiration includes soil microbial and root respiration during the growing season, whereas soil respiration is just derived from microbial organisms after the wheat harvest. The unbalanced temperature and precipitation distributions on the Chinese Loess Plateau further complicate the soil respirational responses between the hot-wet growing season and the cold-dry non-growing season. However, there have been few systematic investigations on the sensitivity of soil respiration to water and temperature conditions between seasons.

Furthermore, the maximum temperature on the Chinese Loess Plateau is predicted to rise 0.7 to 2.2°C and the minimum temperature to rise by1.2 to 2.8°C during 2010–2039 [20], meanwhile the annual rainfall is very likely to steadily decrease, yet more frequently with heavy rains [21, 22]. Such future climate conditions are very likely to further accentuate the sensitivity of soil respiration to water and temperature changes. Therefore, it is of essential necessity to systematically understand the potential effects of N fertilization to the sensitivity of soil respiration to climate change, so as to have a better management for regional agro-ecosystem on the Chinese Loess Plateau.

In this study, we measured the soil respiration, temperature and moisture, and aboveground biomass under different N fertilization rates in winter wheat systems in the semi-arid Loess Plateau from July 2008 to June 2013. The main goals of this study were to (1) quantify the variations in soil respiration and *Q*_10_ values and (2) identify the factors influencing *Q*_10_ values and soil respiration for different N fertilization rates in a winter wheat system.

## Materials and Methods

### Ethics statement

There were no specific permits required for the described field studies. We confirmed that the site was not privately owned or protected in any way. The field studies did not involve endangered or protected species.

### Site description

This study was conducted on a long-term experimental site established in 1984. The study site was in an upland field of the Changwu State Key Agro-ecological Experimental Station in Changwu (E107°40′, N35°12′, altitude 1,220 m), Shaanxi, China, where winter wheat has been cropped for at least 30 years prior to the experiment. It is located in a region that is typical of the Loess Plateau highland region of northwest China. The soil at the site is a loam (Cumulic Haplustoll; USDA Soil Taxonomy System) developed from loess deposits. The main physical and chemical characteristics of the top soil layer (0–20 cm) at the start of the experiment (1984) were as follows: clay (< 2 μm), 24 g kg^−1^; water holding capacity, 0.29 cm^3^ cm^−3^; bulk density, 1.3 Mg m^−3^; pH, 8.4; total carbonate, 105 g kg^−1^; organic C, 6.5 g kg^−1^; total N, 0.80 g kg^−1^; total P, 0.61 g kg^−1^; Olsen-P, 4.7 mg kg^−1^. After more than 20 years N applications, some characteristics of soil and crop [23, 24] were shown in Table 1.

**Table 1:** Selective soil properties of top layer (0–20 cm) and root C:N ratios of the five N fertilized treatments at beginning of the study (2008).

Treatment	SOC (g kg^−1^)	Total soil N (g kg^−1^)	Soil C/N	Root C/N	Nitrate-N in mature period (g kg^−1^)	Nitrate-N in non-growing season (g kg^−1^)
**N0**	6.50	0.78	8.33	34.64	1.94	1.38
**N45**	7.00	0.83	8.43	28.47	2.34	1.78
**N90**	7.17	0.86	8.36	24.96	2.83	1.93
**N135**	7.31	0.90	8.12	17.93	3.26	2.11
**N180**	7.35	0.91	8.08	17.63	3.19	2.26

Note: SOC represents soil organic carbon; TSN represents total soil nitrogen.

**Fig 1:**
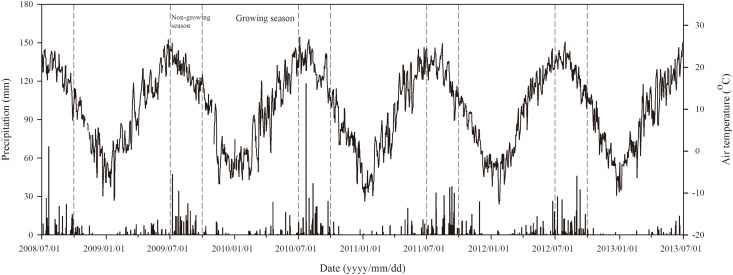
Variations in precipitation (mm) and air temperature (°C) during the experimental period from July 2008 to June 2013.

The current study on the effects of N fertilization to soil respiration rates was started from July 2008 and lasted until June 2013. Winter wheat (*Triticum aestivum* L., cv. Changwu89-134) was sown in late September the first year and harvested in late June the following year. Therefore, we defined from October the first year to June the following year as growing season, and July to September as non-growing season. During the five years of observation, the distribution of precipitation and air temperature was representative of local climate, with the mean annual air temperature of 10.0°C, and the mean annual precipitation of 528.5 mm (ranging from 403.0 mm to 666.8 mm) (Fig 1). As a typical climate pattern on the Loess Plateau, the natural precipitation is peculiarly unsynchronized with the growing seasons of winter wheat: 58% falls during the short term (three months) non-growing seasons, and 42% falls in the long term (nine months) growing season. Such unbalanced soil temperature and moisture distributions between the non-growing and growing season made it necessary to separately consider their individual performances on soil respiration and hence *Q*_10_ values.

### Experimental design

The 15 plots (6 × 4 m) were divided and marked as five different N fertilizer treatments with three replications each in a complete randomized block design. The plots were separated by a 0.5 m wide buffer strip with 1 m strips between adjacent blocks to minimize the disturbance from neighboring treatments. N fertilizer (urea) was applied annually at rates of 0, 45, 90, 135, and 180 kg N ha^−1^ (termed as N0, N45, N90, N135, and N180, respectively). All of the plots received basal phosphate fertilizer at a rate of 39 kg P ha^−1^. All of the fertilizers were applied in a single dose 5–7 days prior to sowing [25, 26]. The aboveground biomass, including the straw and grain, was harvested manually by cutting close to the ground (roughly with residue height of 2 cm), and all harvested biomass was removed from the plots at physiological maturity each year. Winter wheat in eight rows covering one third of the total area of the plot was weighed after air drying, and samples were then collected [25, 26]. In each plot, in order to generate minimum soil compaction and disturbance, only one collar was set up. When measuring the soil respiration rates at each collar, to ensure data reliability, consecutive measurements were carried out until the variation of replicates was smaller than 15%.

### Measurement of soil respiration, temperature and moisture

The soil respiration rates were measured using an automated closed soil CO_2_ flux system equipped with a portable chamber (20 cm in diameter, Li-8100, Lincoln, NE, USA). Approximately one day before the first measurement, a polyvinyl chloride (PVC) collar (20 cm in diameter by 12 cm in height) was inserted 10 cm into each plot. All visible living organisms were removed before the measurements. The final soil respiration values for a given collar were calculated as the mean values of two consecutive satisfactory measurements with a 30 s delay between measurements. The measurement time for each collar was 150 s, which included a 30 s pre-purge, 30 s post-purge, and 90 s observation period.

The measurements were performed between 9:00 and 11:00 local time on each sampling day from July 2008 to June 2013. Sampling frequency has been considered according to the weather conditions. As a rain-fed area with no irrigation, we intentionally stayed with approximately once every two weeks during the dry and prolonged growing season (except for months when the soil was frozen), and increased the sampling frequency to once about 10 days during the wet and short non-growing season. Meanwhile, additional measurement was carried out after particular rainfall event during the wet non-growing season to best capture any possible peaks in soil respiration. Even so, the accumulative annual respiration may also have biases, more likely to underestimate by losing some small peaks. This would only make our estimation even more conservative. In addition, such possible biased situation was supposed to be consistently experienced by all the five treatments. All the five treatments were planted with the same crop (winter wheat) by identical tillage practices.

The soil temperature and volumetric soil moisture measurements were performed concurrently with the soil respiration measurements, adjacent to the chamber placement at a depth of 5 cm. The soil temperature was measured using a Li-Cor thermocouple probe and the soil moisture was determined using the Theta Probe ML2X with an HH2 moisture meter (Delta-T Devices, Cambridge, England). The soil water-filled pore space (WFPS) was calculated by the following equation: WFPS (%) = [volumetric water content / 100 × (2.65 − soil bulk density) / 2.65].

### Crop sampling

The grain yields were determined at maturity by harvesting the central eight rows of the plots. The grain samples were air-dried on concrete, threshed, and then oven-dried at 60°C for 48 h to a stable moisture level and then weighed to estimate the grain yield.

### Data analysis

#### Soil respiration data

The mean soil respiration rate from each N treatment on a certain measurement day was averaged from all the repeated measurements at each collar of the three replicates.

#### Grain yields

The grain yield is the average of 5 years (2009 to 2013).

#### Relationship between soil respiration rate and temperature

An exponential function was used to simulate the relationship between the soil respiration rate and temperature [27]:

$$Rs=\beta_{0}e^{\beta_{1}T}$$ (1)

where Rs (μmol m^-2^ s^-1^) is the measured soil respiration rate; T (°C) is the measured soil temperature at a depth of 5 cm; and *β*_0_ and *β*_1_ are regression coefficients.

#### *Q*_10_ calculation

The temperature sensitivity (*Q*_10_) of soil respiration, which is the multiplier for the soil respiration rate for a 10°C increase in temperature, was calculated as follows [28]:

$$Q_{10}=e^{10\beta_{1}}$$ (2)

where *β*_1_ is obtained from Eq (1).

#### Statistical analysis

The data for the soil respiration rate, temperature and moisture were processed using an Excel 2007 spreadsheet. Performed in SPSS 17.0 for Windows, a one-way ANOVA, followed by the least significant difference test, was used to estimate the difference of *Q*_10_ among the fertilizer treatments in each year at the 0.1 probability level, grain yield and soil respiration over five years at 0.05 probability level. A repeated measures ANOVA was used to estimate the difference of *Q*_10_ between growing season and non-growing season at 0.1 probability level.

## Results

### Effects of N fertilization rates on grain yield

**Fig 2:**
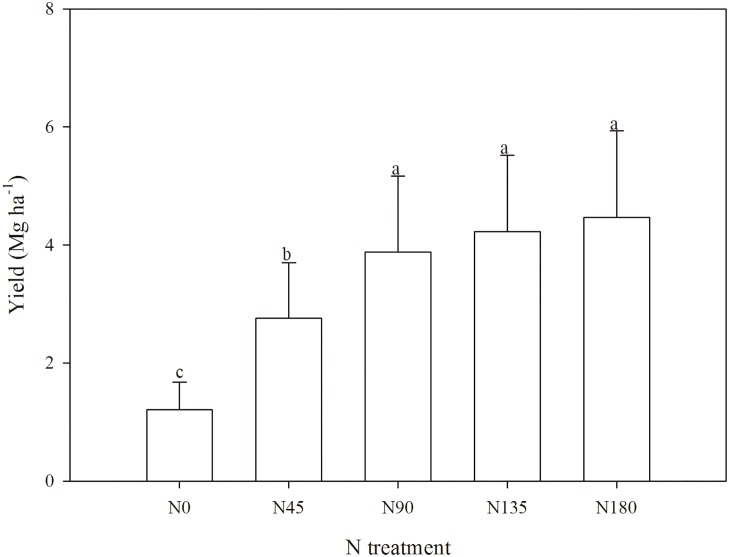
Variations in grain yield (Mg ha^−1^) under different N treatments. The letters (a, b) within the columns indicate the significant differences among the N treatments at the 5% level. Error bars represent standard errors.

The grain yield significantly was increased after N fertilization (*p* < 0.05), yet no significant differences were observed among the N90, N135, and N180 treatments (Fig 2). Compared with the N0 treatment (1.21 Mg ha^−1^), the mean grain yield was 2.28-, 3.20-, 3.49-, and 3.69-times greater in the N45, N90, N135, and N180 treatments, respectively.

### Effects of N fertilization rates on soil respiration rates

**Fig 3:**
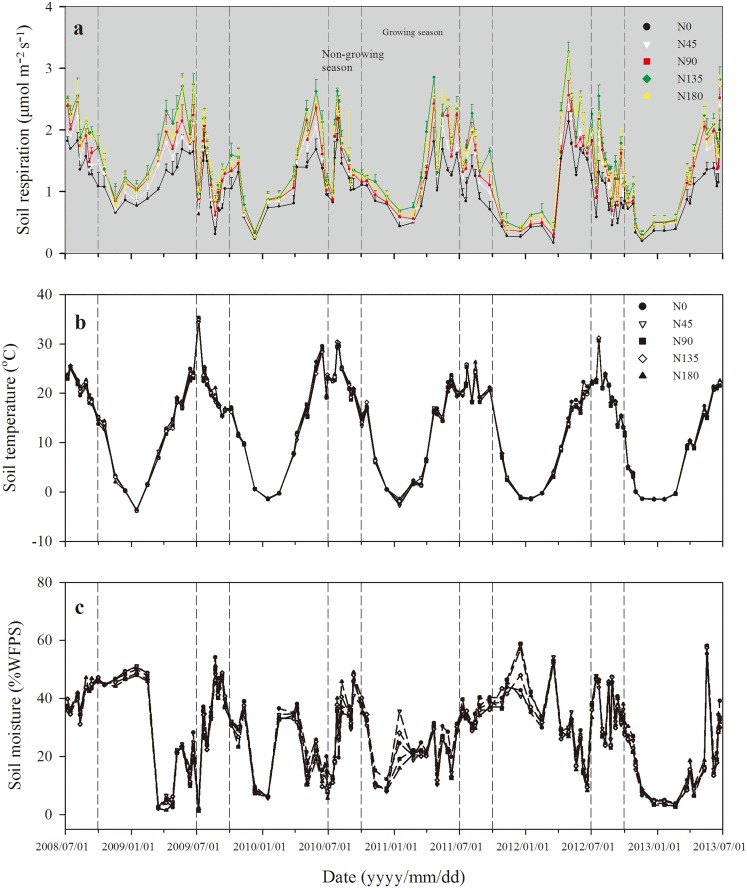
Dynamics of soil respiration rates (μmol m^−2^ s^−1^) (a), soil temperature (°C) (b), and soil moisture (%WFPS) (c) under different N treatments over a five-year period from July 2008 to June 2013.

Compared with the unfertilized treatment (1.07 μmol m^−2^ s^−1^), the soil respiration rate significantly increased under the fertilized treatments (N45-N180) (*p* < 0.05) (Fig 3a). As the N fertilization rates increased, the mean soil respiration rates increased from 1.30 μmol m^−2^ s^−1^ at N45 to 1.49 μmol m^−2^ s^−1^ at N180, with a peak observed in the N135 treatment (1.57 μmol m^−2^ s^−1^).

**Fig 4:**
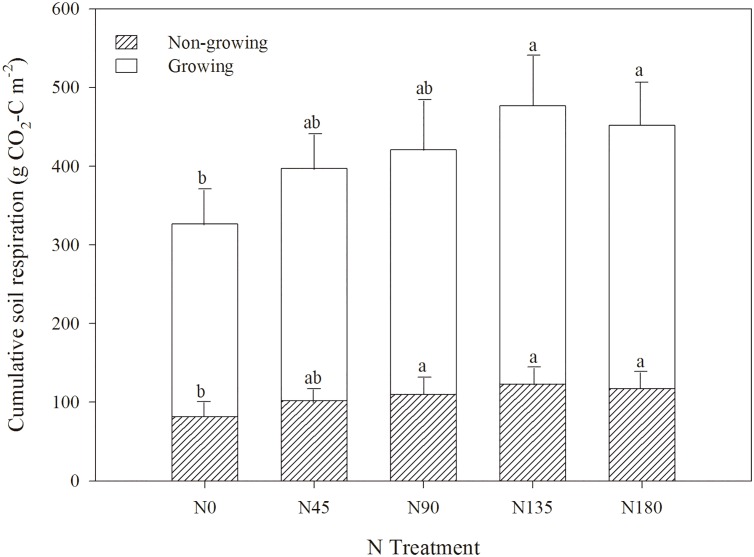
Variations in the cumulative soil respiration (g CO_2_-C m^−2^) under different N treatments during the growing and non-growing seasons. The letters (a, b) within the columns indicate the significant differences among the N treatments at the 5% level. Error bars represent standard errors.

The seasonal variations in the soil respiration rates were similar in all treatments during the study period (Fig 3a), with the highest soil respiration rate before harvest (late April to June), and the lowest soil respiration rate in the cold season (November to mid–March next year). During the study period, the average soil respiration rate for the non-growing season was 1.42 μmol m^−2^ s^−1^ (with a range of 0.32–2.63 μmol m^−2^ s^−1^), which was 7.3% greater than that of the growing season (on average 1.33 μmol m^−2^ s^−1^ with a range of 0.22–3.27 μmol m^−2^ s^−1^). Overall, the cumulative soil respiration increased with increasing N fertilization rates, with a peak observed in the N135 treatment (Fig 4).

### Effects of N fertilization rates on *Q*_10_

**Fig 5:**
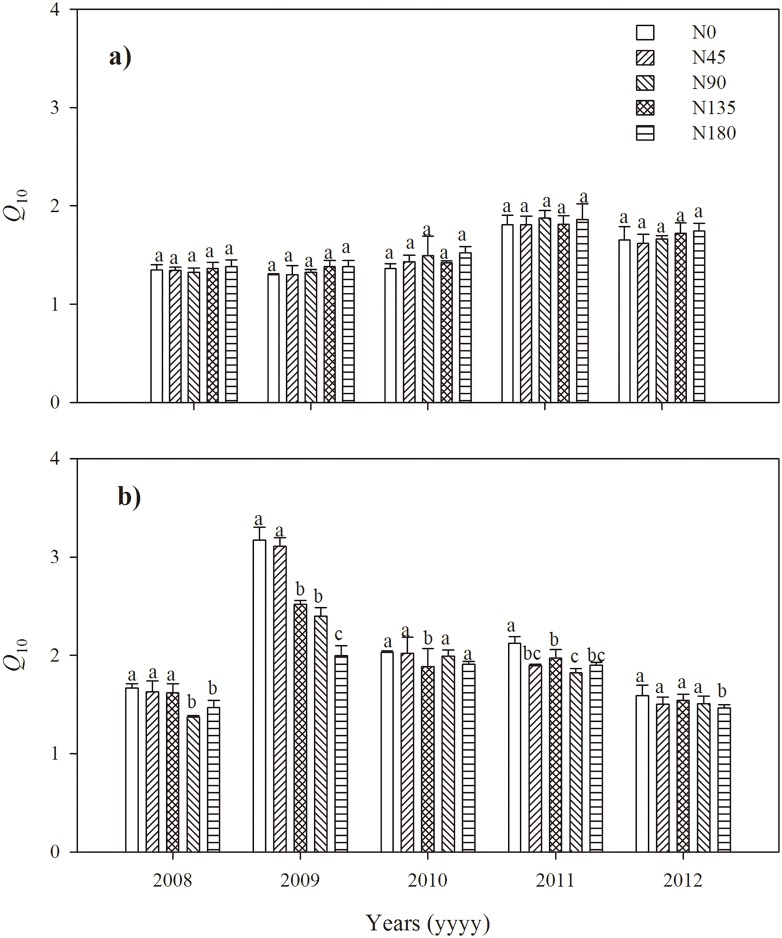
The calculated *Q*_10_ values under different N fertilization rates during growing season (a) and non-growing season (b) over the period of five-year observations. Note the different measures of years: growing season spans from Oct to June next year, while the non-growing season is from July to September of the same year. The low-letters within the columns indicate significant differences among the N treatments at the 10% level. Error bars represent standard errors.

The N fertilization rates had different effects on the *Q*_10_ values between the growing season and the non-growing season (Fig 5a and 5b), and there were also inter-annual variations over the five years. In general, the *Q*_10_ value of the non-growing season was significantly higher than that of the growing season (*p* < 0.1). To be specific, in the growing season (Fig 5a), the *Q*_10_ values did not change significantly with the N fertilization rates, as well as over cultivation years. In the non-growing season, although in varying patterns, the *Q*_10_ values significantly decreased with the N fertilization rates, and year 2009 had exceptionally greater *Q*_10_ values than other years (Fig 5b). In particular, the *Q*_10_ values of the N0 and N45 treatments in the non-growing season were markedly greater than those of all treatments in the growing season (*p* < 0.1).

## Discussion

### Effects of N fertilization rates on soil respiration

The consistent temporal variations of soil respiration (Fig 3a) and soil temperature (Fig 3b) over study period clearly illustrate the decisive effects of temperature to soil respiration. The persistently increasing soil respiration rates (Fig 3a) and cumulative soil respiration amount (Fig 4) with N fertilization rates further demonstrate the enhancing effects of N fertilization to soil respiration activities. This is probably because N fertilization significantly increased the amount of available N (nitrate) in the soil (Table 1), thus promoting the growth of the root system, which enhanced root respiration [16, 29]. N fertilization also improved crop yields (Fig 2), which potentially contributed increasing amount of fresh crop residues for active microbial respiration [30, 31]. However, such enhanced soil respiration performances were not peaked at the greatest N fertilization rate (N180), but at the N135 treatment, indicating that there is possibly a maximum N fertilization rate for the soil to achieve new equilibrium [27]. In fact, as a semi-arid climate with limited natural rainfall and no irrigation, N leaching in winter wheat crop field was small. Previous study based on the same long term N application experiment had shown an accumulation peak of N at 100–180 cm at the N135 and N180 treatments, but no such peaks were observed at the N0, N45, and N90 treatment [32]. Therefore, the accumulation of N in deep layers of great dose of N fertilizer in previous study, as well as the decline of soil respiration at the N180 treatment in this study, jointly suggest that the stimulating effects of N fertilization to root growth and respiration would probably cease after N fertilization rate exceeds above the N135 treatment (135 kg N ha^−1^).

As to different seasons, although the greatest respiration rates were observed before harvest, there were particularly high respiration rates immediately after harvest (Fig 3a) when root respiration was supposed to cease. This was probably because that the time immediately after harvest was coincided with both high temperature and rich precipitation (Fig 3b and 3c), which was exceptionally favorable for microorganism to thrive upon residues and dead roots. To be specific, the average soil temperature for the short non-growing season was 21.5°C (only 11.2°C in the growing season), meanwhile with 58% of the annual precipitation, resulting in 36.9% greater soil moisture content than the growing season (Fig 3b and 3c). Such advantageous temperature and moisture conditions were most effective immediately after harvest, largely promoting the decomposition of microorganisms on the returned fresh residues and roots, thereby dramatically stimulating soil respiration [33, 34]. Once the most easily accessible substrates were gradually consumed, the microbial respiration would then be stabilized down as winter approached.

### Effects of N fertilization rates on *Q*_10_

Unlike the stimulated performance of soil respiration rates, the *Q*_10_ values responded differently to N fertilization during the growing and non-growing seasons (Fig 5a and 5b). In general, the *Q*_10_ values during the non-growing seasons were greater than that during the growing seasons (Fig 5a and 5b). This was probably resulted from the temporal variations of precipitation and air temperature over the growing and non-growing seasons (Fig 1). On one hand, the rich precipitation during the non-growing seasons made the soil moisture (Fig 3c) not anymore the limiting factor for microorganism activities [35]. On the other hand, the generally high temperature (Fig 3b) during the non-growing seasons forced the microorganism community to be selectively active and thus became more sensitive to the changes of soil temperature, therefore leading to greater *Q*_10_ values than the cold and dry growing seasons.

Specifically for the non-growing season where microbial respiration was predominant, the *Q*_10_ values decreased with increasing N fertilization rates (Fig 5b). This is probably related to the gradually decreased C:N ratios of winter wheat roots (0–20 cm) and soil C:N ratios (Table 1) with the increasing N fertilization rates. That means, with the gradually decreasing C:N ratios of soil and root residues, microorganism in treatments of increasing N fertilization rates just required less and less activation energy to decompose abundantly accessed materials [36, 37], consequently resulting in less sensitive changes of respiration rates responding to temperature changes (i.e., smaller *Q*_10_ values). In particular, the considerably greater *Q*_10_ values in year 2009 were probably because the limited precipitation (Fig 1) and the exceptionally greater soil temperature (Fig 3b) during the non-growing season largely intensified the sensitivity of soil respiration rates to the changes of temperature.

Different to the non-growing seasons, the *Q*_10_ values did not change significantly with increasing N fertilization rates during the growing seasons (Fig 5a). This can probably be attributed to the additional yet largely variable root respiration during winter wheat growth. In addition to soil microbial respiration, root respiration also played a big role in soil respiration behavior during the growing seasons. With the increasing N fertilization rates, crop yield was evidently increased (Fig 2), hence promising richer root biomass in the soils of greater N fertilization rates. The stimulating effects of root respiration over temperature increase would therefore be more responsive with increasing N fertilization rates. Such stimulated root respiration over N fertilization rates, if not to overrule, at least tended to counterbalance the behavior of microbial respiration that supposedly required less activation energy in the soils with greater N fertilization rates. In addition, root respiration during the growing seasons was also affected by the crop performance in response to changes in precipitation and soil temperature, which may potentially bring in some unknown variations in *Q*_10_ values. Therefore, further research to specifically quantify the behavior of root respiration and its responses to temperature changes is of great necessity to fully understand the effects of N fertilization to soil respiration.

### Implications

Overall, the observations of soil respiration in this study (Fig 3a) suggest that while high dose of N fertilization could significantly improve crop yield and contributed to regional food security, the potential impacts of widely applied N fertilizer on soil CO_2_ emissions are non-negligible, especially the amount from the non-growing seasons (slightly more than 30% of the total respiration, Fig 4). Furthermore, the inconsistent patterns of *Q*_10_ values during the growing and non-growing seasons (Fig 5a and 5b) suggest that the sensitivity of soil respiration to the changes of soil temperature was not uniform through the year. Such temporal variation of sensitivity to soil temperature may become a great challenge for regional agro-ecosystem management, when confronting the ever-increasing temperature and more erratic rainfall patterns on the Loess Plateau predicted under the future climate conditions [20–22] Therefore, it is essentially necessary to take the effects of N fertilization to soil respiration and the potential variations between seasons into account when modeling the C balances in regional agro-ecosystem.

## Conclusions

This study identified the influence of N fertilization on the *Q*_10_ values of soil respiration during the growing and non-growing seasons under a dry-land wheat cropping system in the semi-arid Loess Plateau. Both soil respiration and *Q*_10_ values varied significantly with N fertilization. As the N fertilization rates increased, the soil respiration rates increased in both the growing and non-growing seasons, whereas the *Q*_10_ values did not change significantly in the growing season but significantly decreased in the non-growing season. However, without planting, the mean *Q*_10_ value during the non-growing season was significantly greater than that during the growing season. Therefore, both the growing and non-growing seasons should be considered when using *Q*_10_ values to estimate soil respiration in agro-ecosystems.

## Supporting Information

S1 File This file contains all Supporting Data (1–5). **Data 1 in S1 File**. Data for Fig 1.**Data 2 in S1 File**. Data for Fig 2. **Data 3 in S1 File**. Data for Fig 3. **Data 4 in S1 File**. Data for Fig 4. **Data 5 in S1 File**. Data for Fig 5.
(RAR)
